# Nutritional Composition, Antinutritional Factors, Antioxidant Activities, Functional Properties, and Sensory Evaluation of Cactus Pear (*Opuntia ficus-indica*) Seeds Grown in Tigray Region, Ethiopia

**DOI:** 10.1155/2019/5697052

**Published:** 2019-05-02

**Authors:** Tewelde Hailemicheal Reda, Mulubrhan Kahsay Atsbha

**Affiliations:** ^1^Police Health Science College, Ethiopian Police University, Addis Ababa, Ethiopia; ^2^Food Science and Post-Harvest Technology, Mekelle University, Mekelle, Ethiopia

## Abstract

Cactus pear (*Opuntia ficus-indica*) seed is one of the main components of fruit crops. The seed is tightly packed together in a mucilaginous structure inside the endocarp of the fruit. The present study investigated the nutritional composition, antinutritional factors, and phytochemical and sensory attributes of cactus pear seeds collected from Hatset Kebele, Hawzen Woreda, Tigray region. The sample provides 392.84 kcal/100g energy in dry weigh basis. The dietary Ca, K, P, Fe, and Zn contents of the sample accounted 390.14mg, 446.46mg, 206.18mg, 4.37mg, and 2.01mg per 100 g, respectively. Despite the high phytate content (259.20mg/100g), the sample had appreciable amount of antioxidant capacity (43 to 95% of inhibition). The sample also had high value of water solubility index (5.6g/100g) and low value of bulk density (0.80g/ml). The sensory evaluation revealed that consumption of “Himbasha” (traditional bread) formulated with wheat flour was more preferable up to the ratio of 85:15% (wheat/seed).

## 1. Introduction

Cactus pear (*Opuntia ficus-indica*) is commonly known as “prickly pear” and grouped under the Cactaceae family [1]. Around 1500 species of cactus are belonging to the* Opuntia* genus among which cactus pear is the most well-known species. Cactus pear can adapt to grow well in wild areas like arid and semiarid regions, where the production of more succulent food plants is severely limited [2]. Most Cactus pears are widely distributed in Europe, Southwestern United States, Northern Mexico, much of Latin America, South Africa, and the Mediterranean countries [3].

Cactus pear, which is locally known by the vernacular name of “Belles”, was introduced to Northern part of Tigray region between 1848 and 1870 by a Catholic Missionary, priest Abune Yakob. He adopted cactus pear with the realization of climatic and topographic unsuitability of the area for cropping and other agricultural activities. Currently, cactus pear is widely spread throughout the region and is believed to cover more than 379,338 hectares of land, i.e., 7.4% of the total land of the region [4]. Only within this region, the uncultivated cactus area covers about 32,000 hectares of land.

Cactus pear is considered as a “Bridge of life” in the region, because its stems and fruits store large quantities of water and provides an important food source to both humans and animals (as forage for livestock). The fruit is also mentioned in traditional song which is translated from the local language (Tigrigna) as ‘A farmer without Belles is like a stream without water.' Cactus pear in Tigray is generally used as a source of food, forage, fuel wood, cash income, live fences, and soil conservation purposes [5]. During summer season, many people especially shepherds do not need to turn back to their home for lunch; instead they are more interested to consume sweet cactus pears.

Since cactus pear fruits have a short postharvest shelf life, it would be essential to prepare in the form of seed flour, juice, and other products which could not be easily perishable throughout the year [6]. The production of seed flours is a simple process (by sun drying or dehydration at low temperature) that changes the discarded seed in to useful and nutritive sources of food [7]. The number of seeds collected from the study area ranged between 290 and 414 or 3.20 and 4.60g per fruit.

Different studies conducted in many countries indicated that cactus pear seed is nutritionally important. However, it is unusual in the study area to use and preserve these seeds as a source of food. Hence, surplus products of cactus pears remain stagnated. Once the ripening period of cactus pear is over, the cultivars tend either to buy their daily foods from the market or they would suffer from starvation during the drought season. This is because the people know nothing about the nutritional and medicinal values of the seed. As far as no study has been conducted in any aspect of cactus pear seed in Ethiopia, the present study intended to determine the nutritional composition, antinutritional factors, antioxidant capacity, and functional properties and to evaluate the sensory acceptability of cactus pear seeds.

## 2. Material and Methods

### 2.1. Sample Collection and Preparation

**Figure 1:**
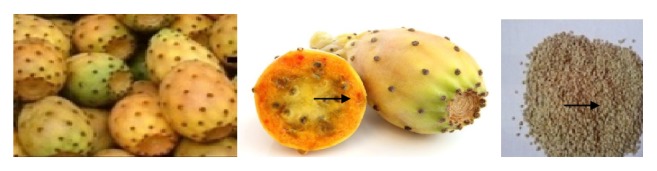
*Opuntia ficus-indica* fruits and their seeds.

Sample was collected and prepared meticulously as indicted in Figure 1. Ripened (yellowish color) cactus pears were randomly collected in October from Hatset Kebele, Tigray region, Ethiopia, and they were washed and peeled using a stainless knife. The seeds were isolated by pressing the whole edible pulp while repeatedly washing with fresh water. The seeds were dried in atmospheric conditions and decorticated using mortar and pistil to remove the sticky-remnant pulps and the seed coat, ground to powder, passed through 0.45mm sieve, and kept in polyethylene bags at room temperature until analysis.

### 2.2. Analytical Methods

#### 2.2.1. Proximate Composition

Moisture, crude protein (N x 6.25), crude fat, crude fiber, and total ash content of cactus pear seeds were determined according to the official method of AOAC [8], while carbohydrate was calculated by difference.

#### 2.2.2. Mineral Content

Zn and Fe contents were analyzed using Atomic Absorption Spectrophotometer (AAS) by the official method of AOAC [9]. Potassium was examined using flame photometer method and phosphorus content was determined by spectrophotometrically using official method of AOAC [8].

#### 2.2.3. Antinutritional Factors

Phytic acid was determined according to the method of Wheeler and Ferrel [10]. Tannin content was analyzed with the method described by Maxson and Roony [11], while oxalate was examined according to the method described by Iwuoha and Kalu [12].

#### 2.2.4. Molar Ratio of Phytate/Mineral

The mole of phytate and minerals was determined by dividing the weight of phytate and minerals with its atomic weight (phytate: 660 g/mol; Ca: 40 g/mol; Fe: 56 g/mol; Zn: 65 g/ mol). The molar ratio between phytate and mineral was obtained after dividing the mole of phytate with the mole of minerals [13].

#### 2.2.5. Phenolic Compounds Extraction and Analysis

Seed flour was extracted using the method of Mau, Chang, Huang, and Chen [14]. A 2.5 g of cactus pear seed flour (duplicate) was weighed, mixed with 25 ml of methanol, and put in incubator shaker at 25°C overnight. The supernatant was decanted into another conical flask and the extraction process was immediately repeated for about 2 hours. The supernatant solution was then poured into weighed rotary evaporator flask to be evaporated using rotary evaporator at 300 rpm and 40°C. After the completion of evaporation, the extract was oven dried at 70°C for further elimination of some vapors and methanol droplets. The dried extract was weighed and the difference in weight was used to remark how much methanol is going to be needed to mix with the dried extract.

Total phenol compound was analyzed calorimetrically, according to the method described by Singleton and Rossi [15]. One ml of cactus pear seed extracts or gallic acid standard solutions was mixed with 1 ml of Folin-Ciocalteu reagent in each test tube, followed by Swire and incubated for 3 minutes at room temperature. After 3 minutes, 1 ml of saturated Na_2_CO_3_ solutions was added and adjusted the solution to 10 ml with distilled water or (add 7 ml distilled water) mixed and incubated at room temperature. The solution was kept in the dark place for 90 minutes. Finally, the absorbance was read at 725 nm using UV-VIS spectrophotometer. The concentration of total phenolic was actually determined using the standard calibration curve of gallic acid at a linearity range of 20-160*μ*g/ml of the curve and values were expressed as milligrams of gallic acid equivalents (mg of GAE/g of dried extract) using gallic acid standard curve.

#### 2.2.6. Flavonoid Compounds Extraction and Analysis

Sample extraction was done according to the method described by Saura-Calixto, Serrano, and Goñi [16]. Briefly 0.4g of dried sample was mixed with 12 ml of acidified methanol water solution (50:50 v/v.pH 2) and extracted for 3 hours. The mixture was centrifuged at 2500 g for 10 minutes and the supernatant was transferred to another test tube. To the residue, 12 ml of acetone water (70:30 v/v) was added and extracted for another 3 hours, centrifugation takes place and the supernatant was mixed with the first extract and stored at 4°C until analysis.

Total flavonoid content was determined using a colorimetric method described by Heimler, Vignolini, Dini, and Romani [17]. 0.25 ml of the seed extract or (+)-catechin standard solution was mixed with 1.25 ml of distilled water in a test tube, followed by adding 75* μ*L of a 5% NaNO_2_ solution. After 6 minutes, 150* μ*L of a 10% AlCl_3_.6H_2_O solution was added and allowed to stand for another 5 minutes before adding 0.5 ml of 1 M NaOH. The mixture was brought to 2.5 ml with distilled water and mixed well. The absorbance was measured at 510 nm using a UV-Visible Spectrophotometer (UV 160, Shimadzu, Japan). The total phenolic content was determined using the standard calibration curve of (+)-catechin at a linearity range of 100-1000*μ*g/ml of the curve and values were expressed as milligrams of (+)-catechin equivalents (mg of CAE/g of dried extract).

The percentage yield extracts were calculated as

(1) $$\begin{array}{c} Yield\left(\;\%\;\right)=\frac{W1}{W2}\times 100 \end{array}$$

where W_1_ is weight of extract after solvent evaporation and W_2_ is weight of the cactus seed flour.

#### 2.2.7. Antioxidant Activity

Methanolic extract (prepared for phenol extract) was also used in this regard. The antiradical DPPH of the seed extract was determined using the method of Kirby and Schemidt [18]. Four ml of 0.004% solution of DPPH radical solution in methanol was mixed with 1 ml of various concentrations (20-240 *μ*l/ml) of sample extract in methanol and was mixed using vortex mixer. The test tube containing the solution was incubated in a dark place for 30 minutes at room temperature. Scavenging capacity was finally read spectrophotometrically by monitoring the decrease in absorbance at 517 nm using U-V and ascorbic acid was used as standard. The scavenging activity was calculated using the following formula:

(2) $$\begin{array}{c} \%\;\;scavenging\;\;activity=\frac{Ac-As}{Ac}\times 100 \end{array}$$

where Ac is the absorbance of the control and As is the absorbance of the sample.

The extract concentration providing 50% of radicals scavenging activity (IC_50_) was calculated from the graph of DPPH inhibition percentage against extract concentration.

#### 2.2.8. Functional Properties

Bulk density was determined according to the method of Narayana and Narasinga-Rao (1984), water absorption capacity (WAC), and water solubility index (WSI) were analyzed according to the method of Sosulski, and McCurdy [19] and Oil absorption capacity (OAC) was determined according to the method of Adeleke and Odedeji [20]. Foaming capacity (FC) and Foaming stability were examined according to the procedure described by Mittal and Kumar [21].

#### 2.2.9. Product Formulation and Sensory Evaluation

Formulation of product was taken between wheat flour and cactus pear seed flour. In order to evaluate the dynamic change in sensory quality of the product, six different formulation ratios of cactus pear seed flour ranged between 0 and 25% and wheat flour were prepared. To each formulation, an equal amount of salt and backing yeast was added. The dough of each formulation was kept at room temperature until floating is started like the preparation of traditional bread “Himbasha”. The bread was then evaluated for its sensory attributes by ten semitrained Food Science and nutrition students. Seven hedonic scales were used to remark the sensory level of each attributes [22]. Each sensory attributes was rated on a nine point hedonic scale (1= disliked extremely while 9 = liked extremely).

#### 2.2.10. Data Analysis

All analyses were carried out in triplicate. Data were analyzed using SPSS (version16) and expressed as means ± standard deviation. Analysis of variance with one factor (ANOVA) was used to determine the difference between the means at 5% level of significance.

## 3. Results and Discussion

### 3.1. Proximate Composition Analysis

To the best of our knowledge, there were no published studies on the nutritional composition and other properties of cactus pear seed grown in Ethiopia though it has been intensively studied in other parts of the world. It has been reported that cactus pear seeds can be used as a source of food since it has high content of protein, fiber, lipids, minerals, and carbohydrates [23]. The study also confirmed that cactus pear seed predominantly contains high amount of carbohydrate followed by fiber (Table 1) and this is in good agreement with the result reported by Nassar [24]. These dietary fibers are important components which may help to prevent a variety of diseases [25]. Cactus pear seed has a fat content (10.50g/100g) which is in harmonious with the findings (10.43g/100g) described by Nassar [24]. The protein and ash content is higher as compared to the result found by Özcan and Al Juhaimi [26]. On the other hand, the fat and protein contents obtained in the present study were highly greater than those values reported by Salim, Abdelwaheb, Rabah, and Ahcene [27]. The difference in results between the present study and other literatures may be due to the variations in climatic conditions, varieties, genetic factors, harvesting time, and soil properties of the land where cactus pears grow.

### 3.2. Mineral Analysis

With regard to the macro- and microelements of cactus pear seeds, appreciable amount of each mineral was obtained in this study (Table 2) as compared to other literatures [28]. In the present study as indicated in Table 2 potassium content was very high (446.46mg/100g) whereas zinc content was relatively low (2.01 mg/100g) as compared to the other minerals and this was in good agreement with the findings of El-Safy, Salem, and Abd El-Ghany [28]. The differences in minerals content reported by various studies could be attributed to the location of plants, application of fertilizers and irrigation use, climate, and genetic differences between the varieties [29].

### 3.3. Antinutritional Factors

Antinutritional factors are generally toxic and may negatively affect the nutritional value of cactus pear seeds by impairing protein digestibility and mineral availability. Three antinutritional factors, namely, phytate, tannin, and oxalate, were examined in this study (Table 3). The results indicated that phytate content was considerably higher (259 mg/100g) than other factors which were similarly reported by El-Safy, Salem, and Abd El-Ghany [28] while the oxalate content was lower than the two antinutritional factors. The presence of condensed tannin compounds is of great importance in the health promotion like the antioxidant components [30]. The antinutritional factors of cactus pear seed can be minimized or eliminated using some processing methods such as soaking [28] and fermenting [31].

### 3.4. Molar Ratio of Phytate to Minerals

The molar ratio between phytate and minerals indicates the impact on the bioavailability of dietary minerals. The critical molar ratio, above which mineral absorption may be inhibited, has been determined as PA:Ca>1.56, PA:Fe>14, and PA:Zn>10 [32]. In this study these limits were employed to predict the bioavailability of minerals. Accordingly, the results revealed that the molar ratios of both phytate:Ca (0.04) and phytate:Fe (4.99) were found to be below the critical limit. This implies that the bioavailability of calcium and iron is not inhibited by the concentration of phytate present in the cactus pear seed flour. However, the molar ratio of phytate to Zn exhibited a high ratio (12.78) which was beyond the stated critical limit. Thus, such value indicates that the bioavailability of Zn in the seeds is inhibited by phytate. Therefore, the risk of Zn inhibition in this regard requires a preferable mode for minimization of the concentration of phytate in the cactus pear seeds.

### 3.5. Total Phenol and Flavonoid Compounds

Antioxidant compounds of natural plants are more prominent with their functional properties to human health. Thus, cactus pear seed as a crop plant needs to investigate its antioxidant compound and antioxidant activity. In the present study, the antioxidant compound particularly total phenol and total flavonoid accounted 90.2 mg/100g and 0.19mg/100g, respectively. It was noticed that the total phenol content was comparable with the findings (48-89 mg/100g) suggested by El-Mostafa et al. [33]. Concerning the total flavonoid content, the values obtained in this study were in line with the result recorded by Chougui et al. [34]. However, differences in total flavonoids may be created due to the variation in geographical origin of the fruits, degree of maturity, extraction protocols, and analytic assays.

### 3.6. Antioxidant Activities

It has been recognized that the total phenol contents (TPC) of plant extract are directly related to the antioxidant activities due to their redox properties. In the present study, antioxidant activity of cactus pear seed was analyzed. The minimum and maximum capacity of scavenging free radicals of the sample was ranged between 43% and 95% and this inhibition capacity was greater than ascorbic acid standard (20%-94.86%). The concentration of extract providing 50% of radicals scavenging activity (IC_50_) was calculated from the graph of DPPH inhibition percentage against the extract concentration. Thus, the IC_50_ of the sample was 1.32 mg/ml and ascorbic acid was 2.4mg/ml. The lower the IC_50_value is, the higher the scavenging potential is. The concentration of the cactus seed flour extract required for the formation of IC_50_ was more likely closer to the ascorbic acid standards but lower than the results recorded by Toure, Bouatia, Idrissi, and Draoui [35]. The differences in antioxidant activity might be associated with the levels of phenolic compounds since the influence of an extract phenolic composition in the antioxidant capacity is a well-known fact [36].

### 3.7. Functional Properties

Functional properties contribute an important role in determining the competitiveness of ingredients or products in the market, as they can impact the sensory, physical, and chemical properties of a food. Some representative attributes such as bulk density, water/oil absorption capacity, foam capacity and stability, and water solubility index were analyzed to evaluate the functional properties of cactus pear seed flour (Table 4). It was observed that the bulk density which influences the amount and strength of packaging materials, energy density, texture, and mouth feel [37] was lower as compared to the other properties. All investigated properties except bulk density were lower than the results reported by El-Safy, Salem, and Abd El-Ghany [28]. The functional attributes of products may vary considerably due to the differences in the raw material, processing, extraction methods, and environmental conditions used during testing.

### 3.8. Evaluation of Sensory Attributes of Cactus Pear Seed Flour Mixed Himbasha

In the present study the sensory characteristics of cactus pear seed particularly its color, taste, aroma, texture, and overall acceptability were evaluated. These sensory attributes are valuable determinant factors for the quality measure of the product (“Himbasha”). The flour ratios are also used to remark the dynamic change in sensory quality of this product. Hence, the results from Table 5 show that there was no significant difference (p>0.05) between 0% sample replacement (control) and 5% sample replacements in all sensory attributes except texture. Based on the sensory scores marked by the panelist, substitution of cactus pear seed up to 15% was appeared to be acceptable with the approximate preference level of ”moderately” or “slightly like” on the given hedonic scales for all attributes. This was directly agreed with the report of Moreno-Álvarez et al. [38] conducted on cactus pear cladode. From the same table, it was noticed that the level of sensory value decreased as the mixed ratio of cactus pear seed increased and this is in line with the suggestions forwarded by Saenz [39]. Surprisingly, Aroma had the highest score values for all attributes and sample ratios, with a representative preference hedonic range of “like” to “moderately like” which is in good agreement with previous studies [38]. The appreciable aroma level might be due to the presence of volatile organic matters, perhaps the fatty or oily components of the cactus pear seeds.

## 4. Conclusion

The present study demonstrated that cactus pear seeds can be used as sources of food in arid and semiarid areas. It contains high amount of carbohydrate and fiber followed by lipid and protein. In addition, cactus pear seed has an appreciable amount of potassium and calcium and low level of antinutritionals (tannin and oxalate) except phytate. The remarkable capacity of scavenging free radicals resulted from the presence of high value of total phenol in the cactus pear seed. Therefore, the seed can essentially be used as good sources of functional food. The functional properties of the sample show that cactus pear seed flour can be moderately competent in the market. Moreover,* Opuntia ficus-indica* seed flours can be consumed by formulating with wheat flours up to a limited ratio.

## Figures and Tables

**Table 1:** Proximate composition of cactus pear seed flour (g/100g) dry weight basis.

Parameters	Contents
Moisture	4.17 ± 0.00
Crude protein	10.00 ± 0.17
Crude fat	10.50 ± 0.50
Crude fiber	18.23 ± 0.00
Total Ash	1.63 ± 00
Carbohydrate	55.47 ±0.44
Total energy	392.84 ± 2.15

*∗* Values are represented as mean ± SD of triplicate analysis.

*∗*Values for proximate composition are expressed as g/100g and for total energy as kcal/100g dry weight basis. Carbohydrate was determined by difference.

**Table 2:** Mineral contents of cactus pear seed (mg/100g, dry basis).

Components	Values
Ca	390.14 ± 0.01
K	446.46 ± 0.01
P	206.18 ± 0.03
Fe	4.37 ± 0.00
Zn	2.01 ± 0.01

*∗* Values are represented as mean ± SD (n=3).

**Table 3:** Antinutritional factors of cactus pear seed (mg/100g, dry basis).

Components	Values
Phytate	259.20 ± 3.700
Tannin	0.13 ± 0.004
Oxalate	0.11 ± 0.09

*∗* Values are represented as mean±SD (n=3).

**Table 4:** Functional properties of cactus pear seed flour.

Properties	Values
Bulk density(g/ml)	0.80 ± 0.00
Water absorption capacity (%)	1.64.00 ± 0.1
Oil absorption capacity (ml)	1.45 ± 0.07
Water solubility index (%)	5.6 ± 0.00
Foaming capacity (ml)	4.75 ± 0.35
Foaming stability(ml)	3.75 ± 0.35

*∗* Values are presented as mean ± SD (n=3).

**Table 5:** Sensory attributes of cactus pear seed and wheat flour ‘Himbasha'.

Flour ratios (wheat: cactus pear seed flour)	Sensory attributes				
	Color	Taste	Aroma	Texture	Overall Accep.
100:0% (control)	8.6±0.97^**a**^	8.1±1.45^**a**^	8.1±0.88^**a**^	8.3±1.25^**a**^	8.3±0.94^**a**^
95:5%	7.7±2.4^**ab**^	7.3±2.01^**ab**^	8.1±0.88^**a**^	6.6±2.59^**b**^	7.1±2.37^**a**^
90:10%	7.1±1.52^**b**^	7.5±1.96^**ab**^	7.9±0.74^**ab**^	6.7±1.40^**b**^	7.3±2.00^**a**^
85:15%	5.8±2.42^**c**^	6.5±2.17^**ab**^	7±1.33^**bc**^	6±2.7b^**c**^	5.5±2.79^**b**^
80:20%	4.4±2.22^**d**^	5.8±1.93^**bc**^	7.1±1.44^**abc**^	5.3±2.45^**cd**^	4.9±1.05^**b**^
75:25%	3.6±2.22^**d**^	4.6±1.78^**c**^	6.7±1.42^**c**^	4.4±2.22^**d**^	4.3±2.49^**b**^

*∗*Values are represented as mean±SD.

*∗*Means followed by the same superscript letter in the same column are not significantly different (p<0.05). The first value in the flour ratios corresponds to wheat flour and the second value to cactus pear seed flour. The values of each sensory attributes represents the hedonic scale (1= disliked extremely while 9 = liked extremely).

## Data Availability

The data used to support the findings of this study are included within the article.
